# Long intergenic noncoding RNA 00673 promotes non-small-cell lung cancer metastasis by binding with EZH2 and causing epigenetic silencing of HOXA5

**DOI:** 10.18632/oncotarget.16158

**Published:** 2017-03-13

**Authors:** Chenhui Ma, Guannan Wu, Qingqing Zhu, Hongbing Liu, Yanwen Yao, Dongmei Yuan, Yafang Liu, Tangfeng Lv, Yong Song

**Affiliations:** ^1^ Department of Respiratory Medicine, Jinling Hospital, Nanjing 210002, China; ^2^ Nanjing University Institute of Respiratory Medicine, Nanjing 210002, China; ^3^ Nanjing Medical University Affiliated Wuxi Second Hospital, Wuxi 214002, China; ^4^ Nanfang Hospital, Southern Medical University, Guangzhou 510515, China

**Keywords:** long noncoding RNA, linc00673, NSCLC, metastasis, HOXA5

## Abstract

Metastasis of cancer cells is a key impediment to favorable outcomes of cancer treatment. Functional roles of long noncoding RNAs in several biological processes, including metastasis, have recently been discovered. In our previous work, we reported a positive correlation of increased expression of linc00673 in NSCLC tissues with tumor size, lymph node metastasis, TNM stage, and increased proliferation of NSCLC cells, both, *in vitro* and *in vivo*. In this study, we demonstrate that ectopic expression of linc00673 promotes migration and invasion of NSCLC cells. Furthermore, our results indicate that linc00673 could silence HOXA5 expression by recruiting epigenetic repressor, EZH2, at its promoter regions. HOXA5 was identified as a tumor suppressor gene, which inhibited NSCLC cell metastasis by regulating cytoskeletal remodeling. To summarize, we for the first time identified the role of lin00673 in promoting invasion and migration of NSCLC cells. Insights from this study may help to identify novel therapeutic targets for NSCLC.

## INTRODUCTION

Lung cancer is characterized by malignant traits such as tumor heterogeneity, aggressive proliferation, a high propensity for distant metastasis, and associated metabolic disorders. Despite advances in diagnostic and therapeutic approaches [1], many patients inevitably progress to advanced cancer stages with solitary or multiple metastatic lesions. The primary infiltration and secondary transfer of cancer cells are important factors of poor prognosis and failure treatment. The low 5-year survival rate of 16.6% in these patients is largely attributable to metastatic lesions [2]. Therefore, it makes sense to study the underlying molecular mechanisms that contribute to aggressive behavior of lung cancer, and may help develop more effective therapeutic approaches.

The development of distant metastases is a multi-step process mediated via several initiation factors and sustainers, which alter the morphology and mobility of cells, transform the extracellular milieu and help evade the host immune mechanisms, eventually leading to colonization of secondary organs. Previous studies have largely focused on the protein-coding genes which could influence every aspect of cancer cell metastasis. However, with the development of genome-wide sequencing and bioinformatics analysis, long non-coding RNAs (lncRNA) have recently acquired a center-stage in cancer research. Long non-coding RNA refers to a group of RNAs with length of > 200 base apirs (bp) and little protein-coding potential [3]. Several recent studies have suggested an important role of lncRNAs in mediating tumorigenesis and tumor progression [4–7]. For example, MALAT1 (metastasis-associated lung adenocarcinoma transcript 1), a metastasis-related lncRNA universally expressed in a series of human cancers, was shown to promote migration and invasion of cancer cells [8]. LncTNA-LET was generally downregulated in a variety of tumors and involved in hypoxia-mediated metastasis [9]. Upregulated lLncRNA HNF1A-AS1 could regulate NSCLC metastasis by adjusting E-cadherin expression [10]. Therefore, wider and further exploration of the expression patterns and molecular mechanisms of lncRNAs may unravel novel targets to prevent proliferation and dissemination of malignant tumor cells.

Long intergenic non-coding RNA 00673, a 2275bp lncRNA encoded by human chromosome 17q25.1, lacks protein coding potential. A study reported the overexpression of linc00673 in lung adenocarcinoma [11] and our previous research validated this finding in 80 paired NSCLC tissue specimens [12]. Our preliminary assessment of the biological significance of linc00673 revealed its involvement in proliferation of NSCLC cells. Our analysis of the clinicopathological parameters indicated a close relationship of high linc00673 expression with lymphatic metastasis and advanced TNM stages [12]. Therefore, we suspected that linc00673 could alter metastatic fate of cancer cells. In this study, we found that down-regulation of linc00673 attenuated the migratory and invasive potential of NSCLC cells, both, *in vitro* and *in vivo*. With RNA sequencing and qRT-PCR analysis, HOXA5 was found to be the downstream target gene that regulated the metastasis of NSCLC cells [13, 14]. We found that linc00673 repressed the expression of HOXA5 by binding with EZH2.

## RESULTS

### Linc00673 promotes migration and invasion of NSCLC cells *in vitro*

**Figure 1:**
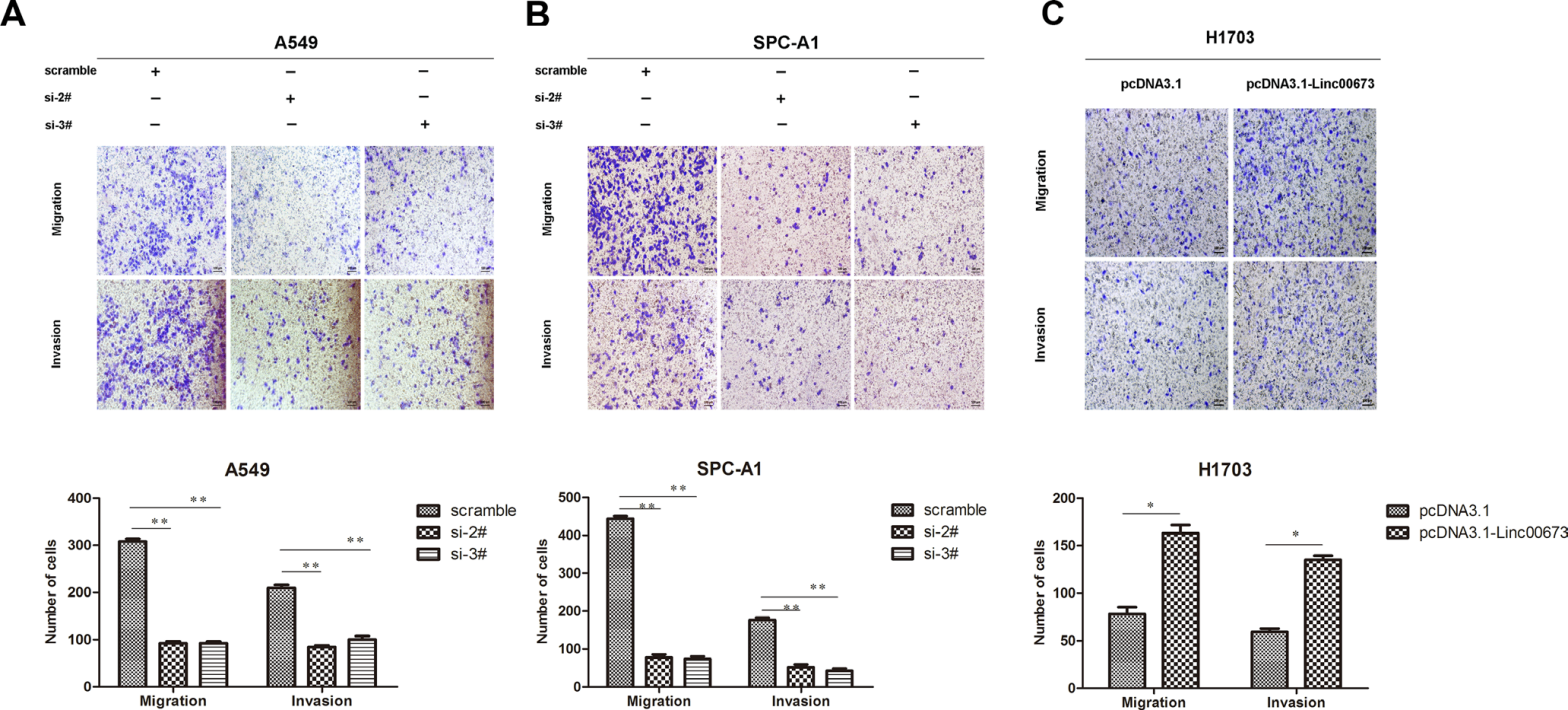
The effect of linc00673 on NSCLC mobility *in vitro* (**A, B**) A549 and SPC-A1 were transfected with si-2# and si-3#. (**C**) H1703 was transfected with pcDNA3.1-linc00673. Transwell assay was conducted to detect change in migration and invasion ability. The data represent the mean ± s.d. of three independent experiments. *P < 0.05, **P < 0.01.

As linc00673 was shown to promote proliferation NSCLC cells, both, *in vitro* and *in vivo*, we further investigated its impact on metastasis. High linc00673 expression cell lines, A549 and SPC-A1, and low linc00673 expression cell line H1703 were chosen for the experiment (Supplementary Figure 1A). Small interference RNA (siRNA) and pcDNA3.1-linc00673 vector were constructed to modulate the expression of linc00673 (Supplementary Figure 1B and Figure 1C).

Transwell assay was performed to assess the biological effect of linc00673 on the mobility of NSCLC cells. Compared with the control group, the number of cells which migrated through the chamber, and the number of cells which invaded through the matrigel were both decreased in A549 and SPC-A1 linc00673-knockdown groups (Figure 1A and Figure 1B). Further, upregulation of linc00673 expression augmented the migratory and invasive abilities of H1703 cells (Figure 1C). These results indicated that linc00673 could promote cancer cell migration and invasion.

### Knock-down of linc00673 inhibits NSCLC cells metastasis *in vivo*

**Figure 2:**
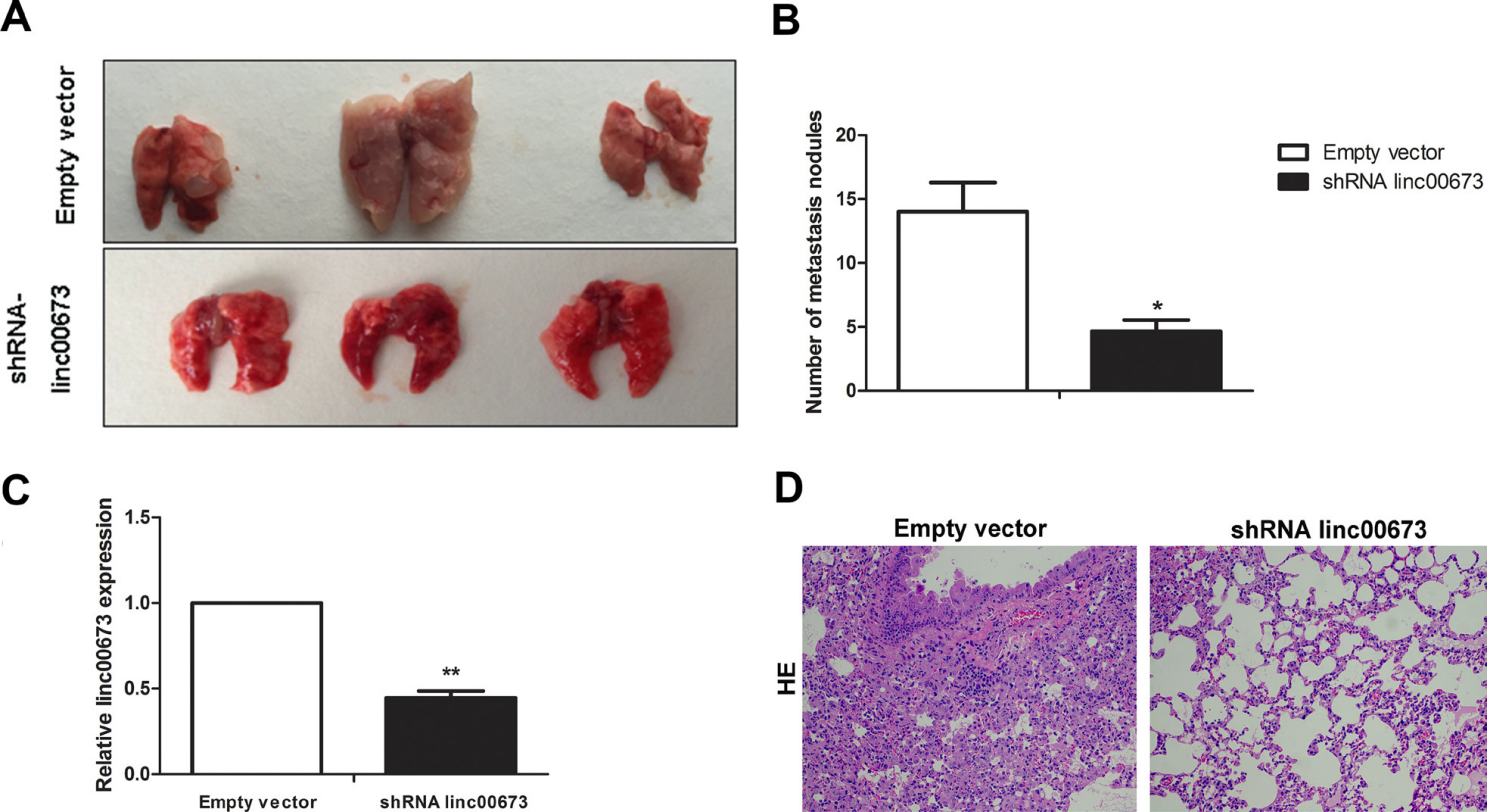
The effect of linc00673 on NSCLC metastasis *in vivo* (**A, B**) SPC-A1 cells were stably transfected with empty vector or shRNA lin00673 and injected into male nude mice via the tail vein as described in the Materials and Methods Section. Lungs were removed from the mice and metastatic nodules counted. (**C**) linc00673 expression levels in lungs of shRNA linc00673 or empty vector transfected SPC-A1 cells on qRT-PCR. (**D**) Representative images (×200) of HE staining. The data represent the mean ± s.d. of three independent experiments. *P < 0.05, **P < 0.01.

To replicate the *in vitro* findings *in vivo*, SPC-A1 cells that were stably transfected with empty vectors or shRNA-linc00673 were injected into 4-weeks-old male nude mice through the tail vein. Seven weeks after injection, the lungs were removed and the metastatic nodules counted. As expected, knock-down of linc00673 expression resulted in a dramatic reduction in metastatic nodules relative to that observed in the control group (Figure 2A and Figure 2B). In addition, the linc00673 expression levels in shRNA linc00673 transfected group were lower than those in empty vector transfection group (Figure 2C).

There were differences between the two groups with respect to hematoxylin and eosin (HE) staining (Figure 2D). These results demonstrated the role of linc00673 in the promotion of cancer cell metastasis, which prompted us to further investigate the underlying molecular mechanisms.

### Gene expression profiling

**Figure 3:**
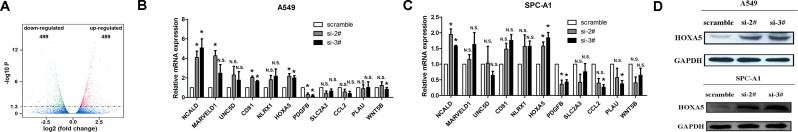
linc00673 regulate the mRNA and protein levels of HOXA5 (**A**) RNA transcriptome sequencing analysis was performed to analyze gene expression profile in A549 cells following linc00673 knock-down. Volcano plot showed all differentially expressed genes. (**B** and **C**) mRNA expression levels of a panel of tumor suppressor genes and oncogenes in control (scrambled) *vs*. si linc00673 (si-2# and si-3#) -transfected NSCLC cells on qRT-PCR analysis. (**D**) Change in HOXA5 protein levels in A549 and SPC-A1 when transfected siRNA-linc00673 on Western blot. The data represent the mean ± s.d. of three independent experiments. *P < 0.05, **P < 0.01.

In our previous study [12], RNA transcriptome sequencing identified 988 differentially expressed transcripts (499 downregulation transcripts and 489 upregulation transcripts, |log2(FoldChange)| > 1 and *P* < 0.05) between linc00673 down-regulated cells and control cells (Figure 3A). Through qRT-PCR, we determined the expression of a panel of representative tumor suppressor genes and oncogenes in A549 and SPC-A1 cells (Figure 3B and 3C). Besides NCALD, HOXA5 was found to be the only metastasis-related gene which had significantly higher mRNA expression in both si-2# and si-3# cells. Furthermore, its protein levels were also found to be correspondingly elevated (Figure 3D).

### Linc00673 silences HOXA5 transcription by binding with enhancer of Zeste Homolog 2 (EZH2)

**Figure 4:**
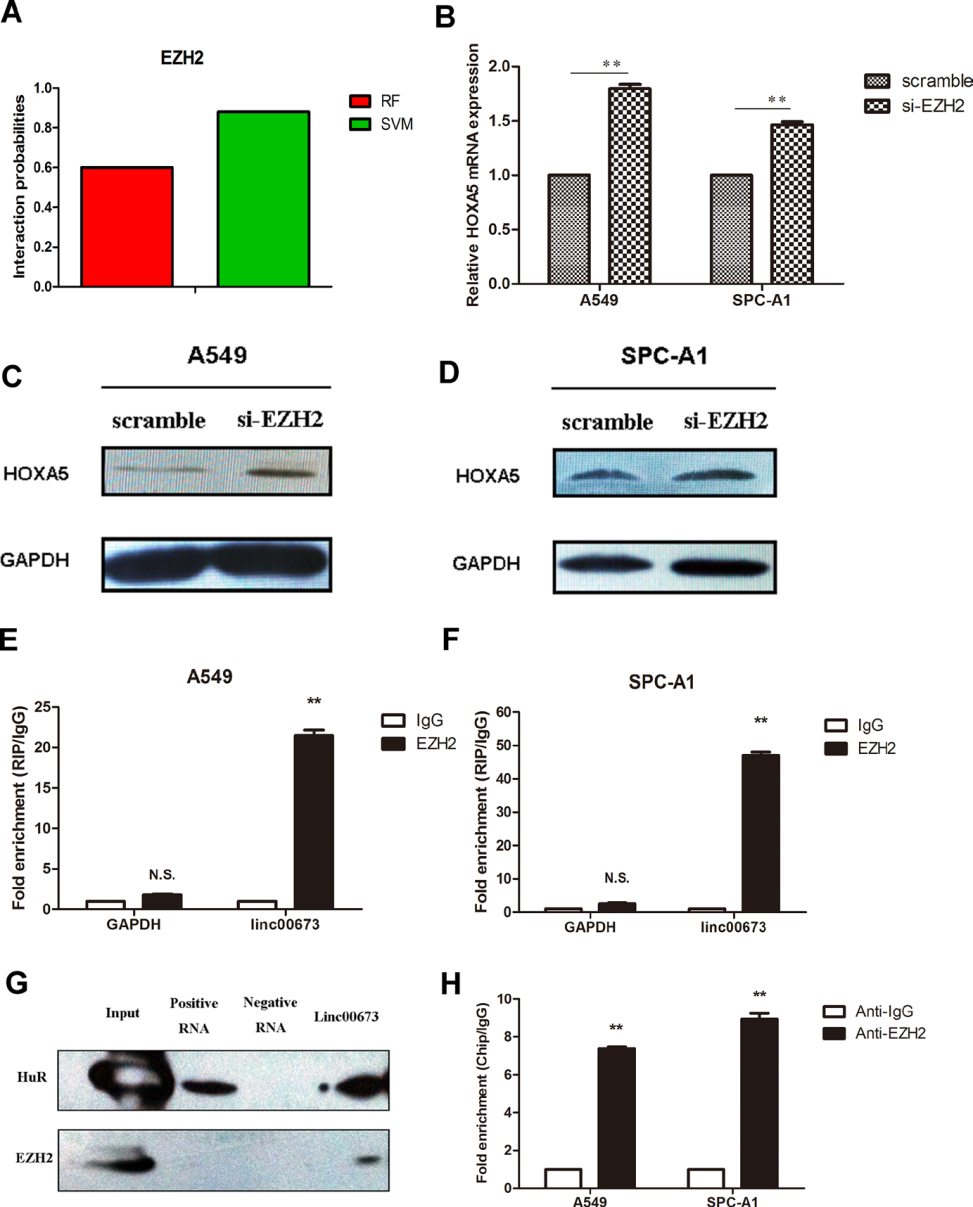
Binding of linc00673 to EZH2 repressed HOXA5 transcription (**A**) The interaction probability of linc00673 with Enhancer of Zeste Homolog 2 (EZH2) was predicted by RNA-Protein interaction prediction website (http://pridb.gdcb.iastate.edu/RPISeq/). (**B, C** and **D**) mRNA and protein changes of HOXA5 in A549 and SPC-A1 when transfected with siRNA-EZH2 on qRT-PCR and Western blot. (**E, F**) RNA binding protein immunoprecipitation (RIP) assay with rabbit monoclonal anti-EZH2 and preimmune IgG from A549 and SPC-A1 cell extracts. Expression levels of linc00673 RNA were presented as fold enrichment in EZH2 relative to IgG immunoprecipitates on qRT-PCR analysis. (**G**) Western blot analysis showed that linc00673 could pull down the EZH2 protein. HuR protein acted as a positive control. (**H**) ChIP of EZH2 occupancy in the NCALD promoter in A549 cells. The data represent the mean ± s.d. of three independent experiments. *P < 0.05, **P < 0.01.

Several studies have shown that lncRNAs and RNA binding proteins (RBPs) synergistically regulate expression of downstream genes [15, 16]. We further explored whether linc00673 regulates HOXA5 expression with the aid of RBPs. First, we determined the probability of linc00673 and RBPs interaction using the RNA-Protein interaction prediction website (http://pridb.gdcb.iastate.edu/RPISeq/). The probability of interaction of linc00673 with EZH2 was 0.6 using RF classifier, and 0.88 using SVM classifier (Figure 4A).

Next, we performed qRT-PCR and Western blot to assess HOXA5 mRNA and protein expression levels when EZH2 was down-regulated. As shown in Figure 4B, 4C and 4D, the mRNA and protein levels of HOXA5 were both higher than those in the control group. To provide stronger evidence to support this hypothesis, we further carried out RIP and RNA-protein pull down analysis.

RIP assay showed that linc00673 could directly bind with EZH2 in A549 and SPC-A1 cells (Figure 4E and 4F). Moreover, RNA-pull down assay validated that linc00673 could actually bind with EZH2 in A549 (Figure 4G). CHIP assay was performed to clarify the relationship between EZH2 and HOXA5, which showed EZH2 bound to the promoter regions of HOXA5 (Figure 4H). These results indicated that linc00673 repressed HOXA5 expression through binding with EZH2.

### Restoration of HOXA5 partially suppresses cancer cell metastasis, both *in vitro* and *in vivo*

**Figure 5:**
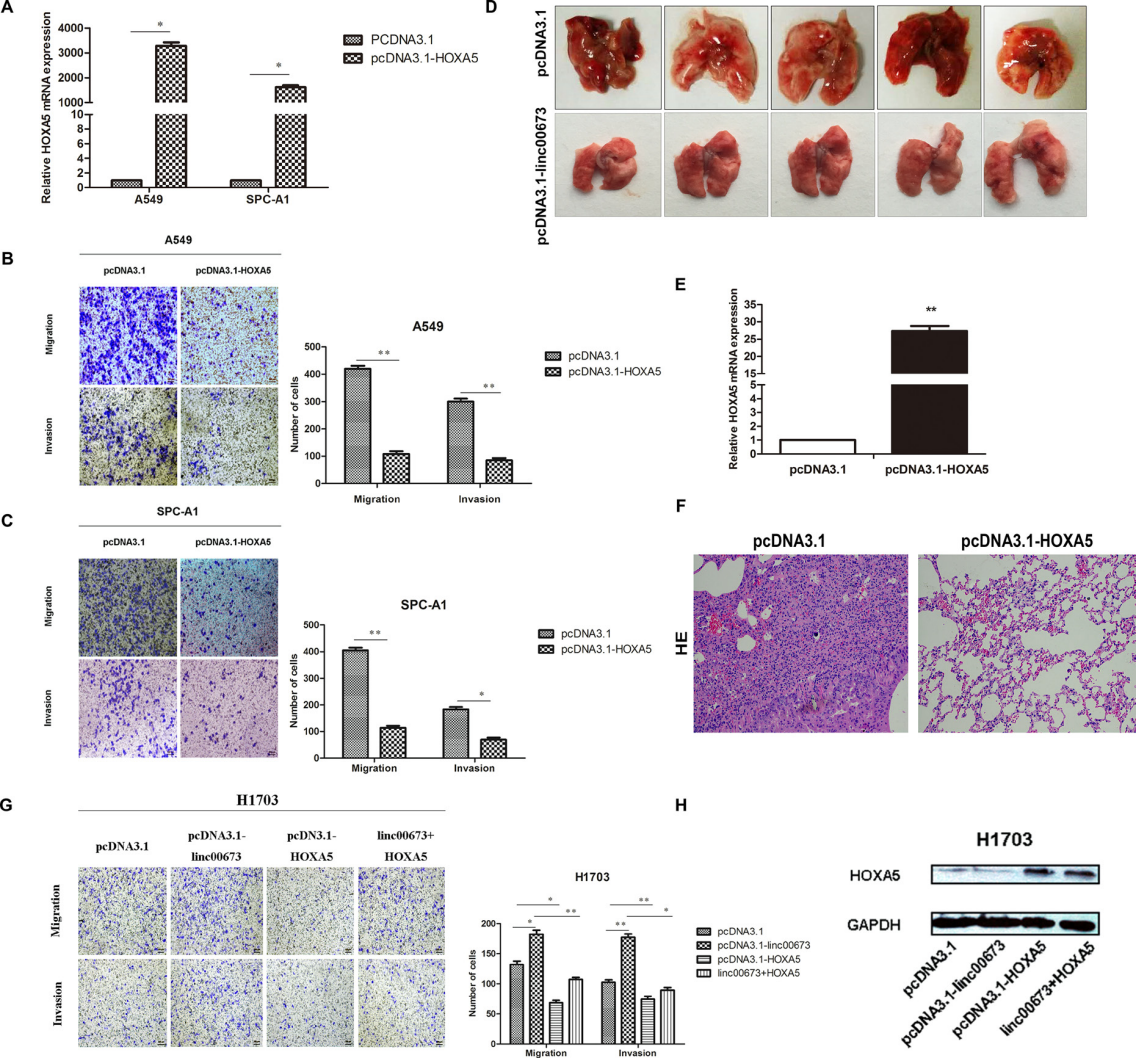
The effect of HOXA5 on NSCLC cells mobility *in vitro* and *in vivo* (**A**) A549 and SPC-A1 were transfected with pcDNA3.1-HOXA5 vector and validated on qRT-PCR. (**B** and **C**) A549 and SPC-A1 were transfected with pcDNA3.1-HOXA5 and transwell assay performed to detect change in migration and invasion ability. (**D**) SPC-A1 cells were stably transfected with pcDNA3.1 or pcDNA3.1-HOXA5 and injected into male nude mice via the tail vein as described in the Materials and Methods Section. Lungs were removed from the mice and metastatic nodules counted. (**E**) HOXA5 expression levels in lungs of pcDNA3.1 or pcDNA3.1-HOXA5 transfected SPC-A1 cells on qRT-PCR analysis. (**F**) Representative images (×200) of HE staining. (**G**) H1703 cells were transfected with pcDNA3.1, pcDNA3.1-linc00673, pcDNA3.1-HOXA5, pcDNA3.1 linc00673 + HOXA5. Transwell assay was performed to determine the cell mobility of transfected NSCLC cells. (**H**) Western blot was performed to determine the HOXA5 protein levels of different transfected cells.

We performed gain-of-function assays to determine the role of HOXA5 in NSCLC metastasis. With qRT-PCR, significant increase of HOXA5 mRNA was observed after transfection with pcDNA3.1-HOXA5 vector, in A549 and SPC-A1 cells (Figure 5A). The protein level of HOXA5 was also increased when transfected with pcDNA3.1-HOXA5 vector in A549 and SPC-A1 cells (Supplementary Figure 2A and 2B). Transwell assay demonstrated that up-regulated HOXA5 in A549 and SPC-A1 prevented cancer cells from migration and invasion (Figure 5B and 5C).

To assess the function of HOXA5 *in vivo*, SPC-A1 cells, which were stably transfected with empty vectors or pcDNA3.1-HOXA5 were injected into 4-weeks-old male nude mice through the tail vein. Seven weeks after injection, the lungs were removed and the metastatic nodules counted. Over-expression of HOXA5 was found to effectively reduce the number of metastatic nodules, when compared with the control group (Figure 5D). Figure 5E shows the different HE staining of the two groups.

In addition, we conducted rescue experiments to test whether restoration of HOXA5 could partially counteract the pro-metastatic effect of linc00673. HOXA5 overexpression partially impaired the linc00673-mediated migration and invasion ability in H1703 cells (Figure 5F and 5G). As presented in Figure 5H, HOXA5 protein levels were upregulated in pcDNA3.1-HOXA5 and linc00673+HOXA5 groups. These findings suggested that compromised HOXA5 function partially reinforced the pro-metastatic effect of linc00673.

### Downregulation of HOXA5 associated with worse clinical parameters in NSCLC

**Figure 6:**
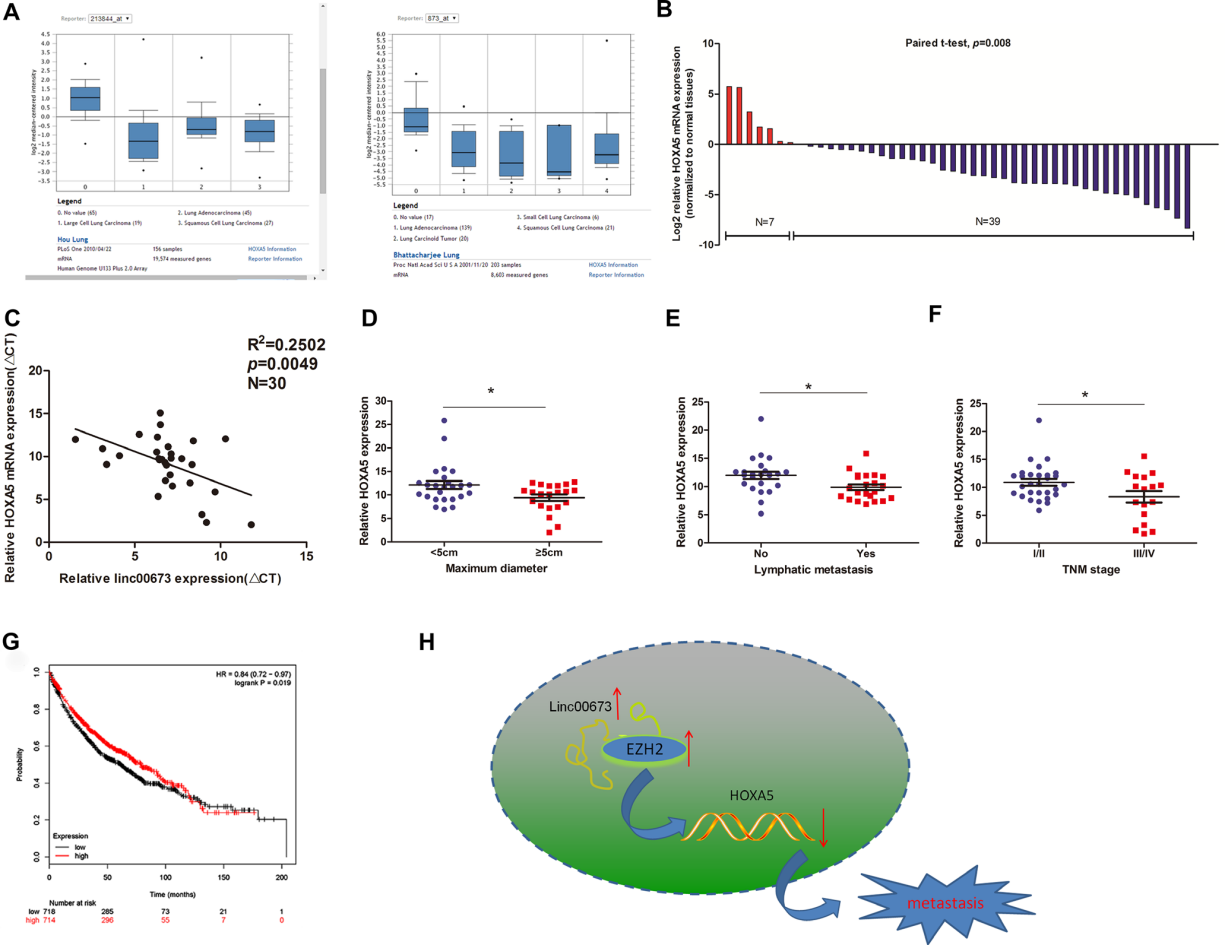
The expression pattern and clinical significance of HOXA5 in NSCLC (**A**) Two microarray datasets from Garber and Bhattacharjee lung cancer cohorts on Oncomine website exhibited the HOXA5 mRNA levels in lung cancer tissues. (**B**) HOXA5 mRNA expression levels in NSCLC tissues on qRT-PCR analysis. (**C**) Correlation of linc00673 and HOXA5 mRNA expression levels in 30 NSCLC tissues (ΔCT value). (**D, E** and **F**) Correlations of HOXA5 expression with clinicopathological parameters. (**G**) Kaplan–Meier survival plots indicated a positive relationship between HOXA5 and overall survival (*n* = 1432). (**H**) Summary diagram describes that linc00673 and HOXA5 regulates NSCLC cell metastasis. *P < 0.05, **P < 0.01.

To exhibit the clinical significance of HOXA5, we initially searched two microarray data sets from Garber and Bhattacharjee lung cancer cohorts on Oncomine website. HOXA5 mRNA expression in NSCLC tissues was found to be significantly lower than that in normal tissues (Figure 6A) [17, 18]. Next, we determined the HOXA5 expression levels in 46 paired clinical cancerous tissues and normal lung tissues. HOXA5 was found to be down-regulated in 39 paired NSCLC tissues and up-regulated in 7 paired tissues (Figure 6B); the findings were consistent with the microarray data. Pearson correlation analysis showed a negative correlation between HOXA5 and linc00673 expression in 30 paired clinical specimens (Figure 6C). Lower HOXA5 expression was associated with larger tumor size, advanced TNM stage and increased lymph node metastasis (Figures 6D, 6E, 6F). In addition, analysis of Kaplan–Meier plots and the associated microarray data of 1432 lung cancer patients (www.kmplot.com) indicated a positive relationship between HOXA5 and overall survival (OS; Figure 6G).

## DISCUSSION

LncRNAs represent an overwhelming amount of transcripts whose biological functions and molecular mechanisms deserve to be explored and expounded in detail [19]. The existing literature indicates unanticipated significant role of lncRNAs in diverse normal as well as pathological processes such as, tissue differentiation, malignant cell proliferation and metastasis [20]. Linc00673 was the most significantly up-regulated lncRNA in human NSCLC tissues, as identified on bioinformatics analysis of GSE19188 and GSE18842. However, a recent study on pancreatic cancer revealed significantly lower expression of linc00673 in cancerous cells and tissues. Further, linc00673 reportedly acted as a tumor suppressor through regulation of the protein tyrosine phosphatase PTPN1 [21]. LncRNA SPRY4 intronic transcript 1 (SPRY4-IT1) was previously reported to be upregulated in melanoma cells., whereafter, Sun et al. found that SPRY4-IT1 expression was downregulated and correlated with a poor prognosis of NSCLC [22]. The contradictory expression patterns and functional roles could be explained by the spatial- and temporal-specific pattern of lncRNAs. In our previous study, we ascertained that linc00673 served as an oncogene in the context of NSCLC, and promoted NSCLC cells proliferation [12]. Furthermore, linc00673 expression was positively correlated with advanced TNM stages and lymph node metastasis. Hence, we hypothesized that linc00673 played an important role in promoting NSCLC cell metastasis. In this report, we investigated its pro-metastasis effect and the associated molecular mechanism. Knock-down of linc00673 inhibited cancer cell migration and invasion, both *in vitro* and *in vivo*. Correspondingly, over-expression of linc00673 enhanced this capacity. These results shed new light on the tumor-promoter role of linc00673.

Considering the limited protein-coding capacity, lncRNAs play an efficient role by regulating downstream target genes, by either directly acting as spong miRNAs or RNA decay, or, indirectly, with the participation of RNA binding proteins at the transcriptional or post-transcriptional levels [23]. Enhancer Zeste 2 (EZH2), a member of the polycomb repressor complex2 (PRC2), acts as an intermediary factor which catalyzes histone H3 lysine 27 methylation to form H3K27me3 during lncRNAs-guided gene silencing [24]. We verified that linc00673 could directly bind with EZH2 in lung cancer cell lines by means of RIP and RNA-pulldown assays, which suggested that linc00673 and EZH2 could regulate downstream genes at the transcriptional level. Further exploration revealed a panel of genes whose expressions were altered alongside the down-regulation of linc00673. On qRT-PCR validation, HOXA5 was found to be a novel candidate gene associated with linc00673. The follow-up ChIP assay confirmed that EZH2 was recruited to the promoter region of HOXA5. To summarize, linc00673 was found to partially repress HOXA5 expression by recruiting EZH2 to its promoter region.

HOXA5 is a tumor suppressive gene which functions as a transcription factor, and was initially reported to be positively regulated by p53 in human breast cancers [25]. Further studies demonstrated that HOXA5 was universally diminished in cancerous tissues and promoted malignant progression by affecting cancer cell proliferation, apoptosis and metastasis [13, 14, 26, 27]. Wang *et al*. reported that HOXA5 inhibited metastasis of lung cancer cells through cytoskeleton-related genes, such as PXN, ARPC4 and PAK1, at the transcriptional level [14]. A recent research found that HOXA5 also played a part in controlling intestinal stem cell traits [28]. In the present study, we found that ectopic HOXA5 could inhibit cancer cell migration and invasion, both *in vitro* and *in vivo*, which is consistent with previous reports [13, 14, 26, 27].

To summarize, in this study, up-regulated expression of linc00673 transcriptionally repressed target gene expression in NSCLC cells by directly binding RBPs, which enhanced cancer cell proliferation and metastasis (Figure 6H). In spite of the discovery of mis-expression of lncRNAs in numerous human diseases, there remain many gaps in our understanding of the complex regulatory elements and molecular mechanisms of non-coding genes. Further studies on lncRNAs should be conducted to generate data on new targets, which may prove to be useful for the diagnosis and treatment of human diseases.

## MATERIALS AND METHODS

### Cell lines

Three cell lines (two lung adenocarcinoma cell lines, A549 and SPC-A1, and one NSCLC squamous carcinomas cell line, H1703) were purchased from the Institute of Biochemistry and Cell Biology, The Chinese Academy of Sciences (Shanghai, China). A549, H1703 cells were cultured in RPMI Medium 1640 basic media (GIBCO-BRL, Invitrogen, Carlsbad, CA), and SPCA1 was cultured in Dulbecco's modified Eagle's media (DMEM; GIBCO-BRL, Invitrogen) supplemented with heat-inactivated 10% fetal bovine serum (FBS) and antibiotics (100 U/mL penicillin and 100 mg/mL streptomycin) (Invitrogen, Carlsbad), in a humidified incubator at 37°C with 5% CO_2_.

### RNA extraction and quantitative reverse transcriptase polymerase chain reaction

Total RNA was isolated from tissues and cells by TRIzol reagent (Invitrogen) according to the manufacturer's instructions. Then, 1 μg RNA was reverse transcribed to cDNA in a final volume of 20 μL by using a Reverse Transcription Kit (Takara, Dalian, China). Real-time PCR was performed using SYBR Premix ExTaq II kit (Takara, Dalian, China) to determine expression levels of linc00673 and HOXA5, normalized to the expression of GAPDH. The PCR primers are shown in Supplementary Table 1. qRT-PCR assay and data collection were performed on ABI 7500, and results analyzed and expressed relative to threshold cycle values (ΔCt), which were then converted to fold changes using the 2−ΔΔCt method. GAPDH was used as an internal control.

### Transfection of lung cancer cells

Linc00673 siRNA and over-expression plasmid were the same as described in the previous study [12]. All plasmid vectors used (pcDNA3.1, pcDNA3.1-linc00673 and pcDNA3.1-HOXA5) were extracted using DNA Midiprep or Midiprep kit (Qiagen, Hilden, Germany). Lung cancer cells were grown on six-well plates to 70%-80% confluence and transfected with siRNA or plasmid using Lipofectamine 2000 (Invitrogen, Shanghai, China) or X-treme GENE HP DNA transfection reagent (Roche, Basel, Switzerland). After 48h incubation, cells were harvested and processed for the following experiments.

### Cell migration and invasion assays

Cells were collected at 48h post-transfection; 5 × 10^4^ (for migration assay) or 1 × 10^5^ (for invasion assay) cells in serum-free media were placed into the upper chamber of an insert (8-μm pore size; Millipore, Billerica, MA, USA). Medium containing 10% FBS was added to the lower chamber. After incubation for 24 h, the cells remaining on the upper membrane were removed with cotton wool; cells which had migrated or invaded through the membrane were stained with methanol and 0.1% crystal violet, imaged, and counted using an IX71 inverted microscope (Olympus, Tokyo, Japan). Experiments were independently repeated three times.

### Western blot assay

Cells were lysed 48h post-transfection, with mammalian protein extraction reagent RIPA (Beyotime China), a protease inhibitor cocktail (Roche, Basel, Switzerland) and PMSF (Roche). The Bio-Rad protein assay kit was used to determine the concentration of protein. 50 μg protein lysates were electrophoresed on 10% SDS-PAGE and transferred onto 0.22 mm NC membranes (Sigma–Aldrich), and then incubated with specific antibodies. ECL chromogenic substrate and densitometry (Quantity One software; Bio-Rad, CA, USA) were used to detect the bands and quantify the intensity respectively. GAPDH antibody was used as control.

### Immunohistochemistry

Paraffin-embedded, formalin-fixed NSCLC tissues and matched normal lung tissues from *in vivo* assays were immunostained for Ki-67 expression using anti-Ki67 antibody (1:50, Abcam). Immunoreaction was performed using the labeled streptavidin-biotin method with overnight incubation. Diaminobenzidine was used for visualization. The intensity and extent of immunoreactivity were evaluated and scored in randomly selected five representative fields of vision at medium magnification. An immunostaining score of 2+ or more was considered positive; a score of 0 or 1+ were considered negative.

### *In vivo* assay

4-week-olds male athymic BALB/c nude mice were sourced from the animal center at the Jinling Hospital. SPC-A1 cells were transfected with pcDNA3.1, pcDNA3.1-HOXA5, shRNA-linc00673 or empty vector and resuspended at a concentration of 2 × 10^7^/mL. For metastasis assays, 0.1 mL of cell suspension was injected into the tail veins of mice (*n* = 7, each group). At 7 weeks post-injection, these mice were sacrificed, the lungs removed and photographed. After counting the visible tumors on the lung surface, the lungs were stored in formalin. Animal care and experimental procedures were approved by the Model Animal Research Center at the Jingling Hospital. All procedures involving use of animals were conducted in compliance with to the Institutional Animal Care and User guidelines. All surgical procedureswere performed under sodium pentobarbital anesthesia, and all efforts were made to minimize suffering.

### RNA-binding protein immunoprecipitation (RIP) assay

Magna RIP RNA-Binding Protein Immunoprecipitation Kit (Millipore, USA) and antibody to EZH2 (Millipore, USA) were used for RNA immunoprecipitation (RIP) experiments. According to the manufacturer's instructions, cells were lysed and incubated with protein A Sepharose beads which were conjugated with antibodies at 4°C. After 3-6 hours, the beads were washed with wash buffer. Then, proteins in the washed complexes were digested through a heated water bath with 0.1% SDS and 0.5 mg/mL Proteinase K (30 minutes at 55°C). Finally, immunoprecipitated RNA was purified and analyzed on qRT-PCR.

### Chromatin immunoprecipitation assays

Chromatin immunoprecipitation assay was performed with EZ-CHIP KIT (Millipore, USA). According to the manufacturer's instructions, first, A549 cells were incubated with formaldehyde to generate DNA–protein cross-links. Then cells were lysed, sonicated and immunoprecipitated with EZH2 (Millipore, USA) or IgG, as control. Finally, precipitated chromatin DNA was recovered and analyzed on qPCR. The sequences of ChIP primers for HOXA5 promoter region amplification are shown in the Supplementary Table 1.

### RNA pulldown assay

linc00673 was prepared *in vitro* by transcribing from vector pcDNA3.1-linc00673. RNA biotin-labeling was performed with the Biotin RNA Labeling Mix (Roche Diagnostics, Indianapolis, IN) and T7/SP6 RNA polymerase (Roche Diagnostics, Indianapolis, IN). RNA purification was carried out using through RNeasy Mini Kit (Qiagen, Valencia, CA). Next, 1 milligram A549 cell lysates and 3 μg purified biotinylated transcripts were mixed and incubated with streptavidin agarose beads (Invitrogen) for 1 h at 25°C. Subsequently, the beads were washed and sodium dodecyl sulfate (SDS) buffer added to the eluent. Finally, the standard Western blot technique was used to analyze the retrieved protein.

### Statistical analysis

SPSS 17.0 software (IBM, Chicago, IL, USA) was used for all statistical analyses; *P* < 0.05 was considered to be statistically significant. Student's *t*-test (two-tailed) and chi-square test analysis were used to analyze differences between groups. Pearson correlation analyses were used to analyze the correlation between linc00673 and HOXA5 mRNA expression.

## SUPPLEMENTARY MATERIALS FIGURES AND TABLES


