# Supplementary material for: Long intergenic noncoding RNA 00673 promotes non-small-cell lung cancer metastasis by binding with EZH2 and causing epigenetic silencing of HOXA5
Source: Oncotarget. 2017 Mar 13;8(20):32696–705. doi: 10.18632/oncotarget.16158 (PMC5464820; doi:10.18632/oncotarget.16158)
Supplement: Supplementary file 1 [file oncotarget-08-32696-s001.pdf]

Long intergenic noncoding RNA 00673 promotes non-small-cell lung cancer metastasis by binding with EZH2 and causing epigenetic silencing of HOXA5

Supplementary Materials

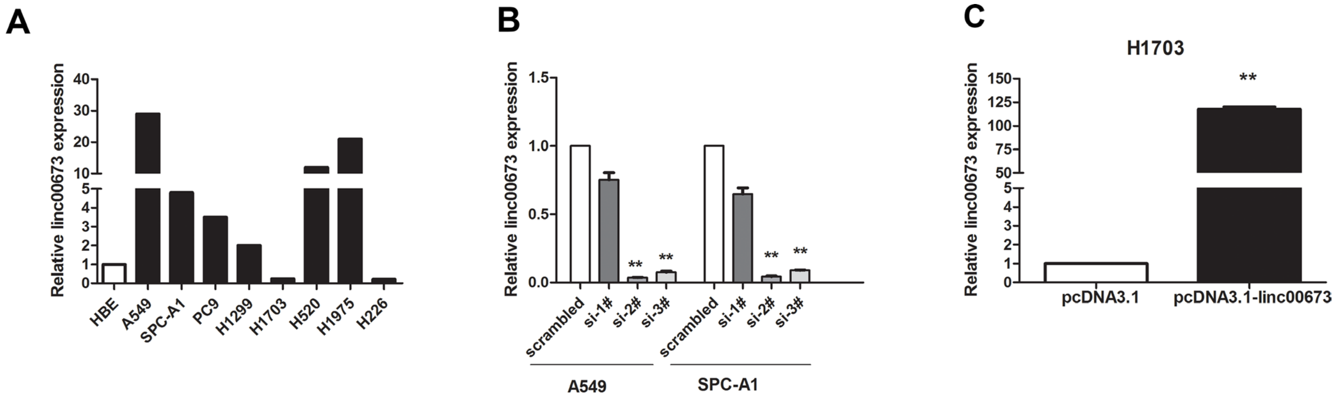

Supplementary Figure 1: The expression levels of linc00673 and the optimization of transfection efficiency. (**A**) Expression levels of linc00673 in 8 NSCLC cell lines and human bronchial epithelial cell (HBE) assessed on qRT-PCR analysis. (**B**) A549 and SPC-A1 were transfected with chemically synthesized siRNA-linc00673. (**C**) H1703 was transfected with pcDNA3.1-linc00673 vector.

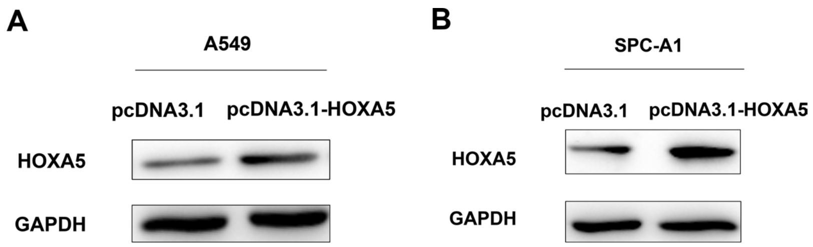

Supplementary Figure 2: The protein level of HOXA5. (**A**) A549 was transfected with pcDNA3.1 and pcDNA3.1-HOXA5. (**B**) SPC-A1 was transfected with pcDNA3.1 and pcDNA3.1-HOXA5.

Supplementary Table 1: primers for qRT-PCR

linc00673	F	TACCACACCCTTTCTTGCCC
	R	ACACTGGCCTCTTTACACGG
HOXA5	F	ATGCGCAAGCTGCACATAAG
	R	CGGGTCAGGTAACGGTTGAA
GAPDH	F	GGGAGCCAAAAGGGTCAT
	R	GAGTCCTTCCACGATACCAA
primers of HOXA5 promotor regions		
P219	F	AAACCTCACACACAGCCAATACC
	R	ACACTAGCACAGGAGCCCCAGAC
P889	F	ATTTGTGATTTACTTGAGTCTTT
	R	GGCGGGTTACTGGGGTCTTGCTT
F3	F	TTGGAGAAACACTACACAAAAGC
F4	R	TTGGAGAAATAAATCCTGCCCGC
